# The Roles of TGF-β Signaling in Cerebrovascular Diseases

**DOI:** 10.3389/fcell.2020.567682

**Published:** 2020-09-18

**Authors:** Yizhe Zhang, Xiao Yang

**Affiliations:** State Key Laboratory of Proteomics, Beijing Proteome Research Center, National Center for Protein Sciences, Beijing Institute of Lifeomics, Beijing, China

**Keywords:** cerebral cavernous malformation, hereditary hemorrhagic telangiectasia, cerebrovascular disease, endothelial-to-mesenchymal transition, cerebral angiogenesis, TGF-β signaling

## Abstract

Cerebrovascular diseases are one of the leading causes of death worldwide, however, little progress has been made in preventing or treating these diseases to date. The transforming growth factor-β (TGF-β) signaling pathway plays crucial and highly complicated roles in cerebrovascular development and homeostasis, and dysregulated TGF-β signaling contributes to cerebrovascular diseases. In this review, we provide an updated overview of the functional role of TGF-β signaling in the cerebrovascular system under physiological and pathological conditions. We discuss the current understanding of TGF-β signaling in cerebral angiogenesis and the maintenance of brain vessel homeostasis. We also review the mechanisms by which disruption of TGF-β signaling triggers or promotes the progression of cerebrovascular diseases. Finally, we briefly discuss the potential of targeting TGF-β signaling to treat cerebrovascular diseases.

## Introduction

According to the latest Global Burden of Disease Study, cerebrovascular disease is the second leading cause of mortality worldwide (Naghavi et al., 2017). Emerging clinical research data show that cerebrovascular disease is also the cause of many central nervous system diseases (Turner et al., 2016; Iadecola and Gottesman, 2018; Kummer et al., 2019). However, due to the lack of techniques to study cerebrovascular development and its regulatory mechanisms at the whole-animal level, our understanding of cerebrovascular diseases is still very limited. Emerging studies have begun to uncover the molecular mechanisms of cerebrovascular development and homeostasis, providing new basis and treatment strategies for the prevention and treatment of cerebrovascular and central nervous system diseases (Vanlandewijck et al., 2018; Munji et al., 2019).

Blood vessels of the brain form a highly specialized vascular network, which have complex interactions with the central nervous system, and has important physiological functions in the development and maintenance of the central nervous system (Zhao et al., 2015; Iadecola, 2017; Paredes et al., 2018). The development of cerebrovasculature begins with the angiogenic sprouting of perineural vascular plexus (PNVP) blood vessels, which forms a delicate hierarchical vascular structure through continued sprouting and remodeling (Tata et al., 2015). Blood vessels of the brain are mainly composed of highly specialized vascular endothelial cells (ECs), which have an arteriovenous differentiation pattern similar to that of peripheral vascular ECs. Brain ECs have obvious heterogeneity, complex tight junctions, more pericyte coverage, and form the neurovascular unit (NVU) together with pericytes, smooth muscle cells (SMCs), astrocytes, and neurons. The brain ECs, pericytes and the endfeet of astrocytes together form the unique blood–brain barrier (BBB) to restrict potentially harmful substances and molecules from entering the brain. The nutrients, energy metabolites, metabolic waste and other essential molecules cross the brain endothelium via various substrate-specific transporters to ensure physiological functioning of the brain. The primitive BBB is formed at embryonic day 15 (E15) in mice and varies in other species (Zhao et al., 2015). BBB continues to mature under the influence of neural environment over a brief period after birth.

Cerebrovascular development is a highly conserved and complex process involving multiple signaling pathways. Using various model organisms, researchers have successively identified many genes and signaling pathways that regulate the formation and homeostasis of blood vessels of the brain, including vascular endothelial growth factor (VEGF), sonic hedgehog/Patched (Shh/PTC-1), platelet-derived growth factor B/platelet-derived growth factor receptor β (PDGFB/PDGFRβ), Wnt/β-catenin, orphan G protein coupled receptor 124 (GPR124), as well as transforming growth factor β/SMAD (TGF-β/SMAD) signaling (Stenman et al., 2008; Ferrari et al., 2009; James et al., 2009; Alvarez et al., 2011; Cullen et al., 2011; Posokhova et al., 2015; Sweeney et al., 2016). As one of the most important and complex signaling pathways in vascular development, TGF-β/SMAD signaling plays diverse functions during the development and homeostasis of the brain vessel, and dysfunction in this signaling pathway has been linked to various cerebrovascular diseases (Park et al., 2009; Nguyen et al., 2011; Maddaluno et al., 2013). In this review, we discuss the latest research progress on the physiological function of TGF-β signaling in cerebrovascular development, and the mechanisms by which disruption of TGF-β signaling causes cerebrovascular diseases.

## TGF-β Signaling in the Development and Homeostasis of Cerebrovasculature

### The TGF-β Signaling Pathway

The TGF-β signaling pathway is highly conserved in evolution, and plays multiple and complex physiological functions in the regulation of embryonic development and tissue homeostasis in a highly context-dependent manner (Morikawa et al., 2016; David and Massague, 2018; Zinski et al., 2018).

**FIGURE 1:**
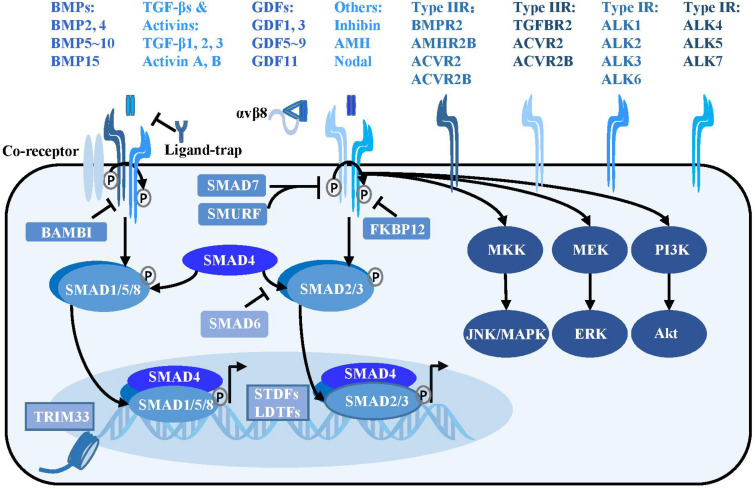
TGF-β signaling pathway. Activated TGF-β ligands with or without αVβ8 integrins bind at serine/threonine protein kinase type II receptors, sometimes with the assistance of the co-receptors such as endoglin. The type II receptors subsequently phosphorylate type I receptors to form a tetrameric receptor complex, which subsequently phosphorylates the SMAD2, SMAD3 or SMAD1, SMAD5, and SMAD8 to form a trimeric complex with SMAD4 in cytoplasm. The SMAD complex then translocates into nucleus and binds at special loci under the guidance of the SDTFs and LDTFs to initiate the transcriptional response. Besides, TRIM33, a modulator of TGF-β signaling, is able to regulate chromatin accessibility and remodeling. In addition to the canonical TGF-β signaling, there are SMAD-independent pathways, such as PI3K/Akt, MEK/ERK and MKK/JNK/MAPK downstream of the TGF-β receptors. TGF-β signaling is negatively regulated at multiple levels. Various ligand traps (noggin, chordin, follistatin, gremlin, coco, and cerberus) can prevent TGF-β ligands from binding to receptors, while FKBP12 and BAMBI can dock at cytoplasmic domain of TGF-β type I receptors to inhibit TGF-β signaling. In addition, inhibitory SMADs including SMAD6 and SMAD7 play a critical function in suppressing the SMAD-mediated signaling.

The TGF-β signaling pathway comprises of more than 30 kinds of ligands, mainly divided into subfamilies such as TGF-βs, bone morphogenetic proteins (BMPs), activins, inhibin, Nodal, anti-Müllerian hormone, and growth and differentiation factors (GDFs) (Figure 1). Most TGF-β ligands function as paracrine factors on adjacent cells. The TGF-β ligands are expressed in latent forms with latency-associated peptide (LAP) shadowing the active domains of TGF-βs in the latent complex, and mature TGF-β ligands are activated through cleavage by extracellular protease from the LAP or physical tension by integrins. Several milieu molecules interact specifically with latent TGF-β and are essential for the bioavailability of TGF-β ligands. It is widely accepted that αVβ6 and αVβ8 integrins convert the cytoskeletal tension into a mechanical force to dissociate LAP from the TGF-β active domain, thereby releasing the activated TGF-β molecule and initiating the signaling cascade (Aluwihare et al., 2009). Very recently, researchers used cryo-electron microscopy to analyze the intermediate conformation of the interaction between αVβ8 integrin and latent TGF-β, and found that latent TGF-β binding with αVβ8 can expose the active domain and directly activate the TGF-β signaling pathway without release of the mature conformation (Campbell et al., 2020).

Activated TGF-β ligands, which are usually disulfide-linked homodimers, directly bind to the serine/threonine protein kinase type II receptors on the cell membrane surface, sometimes with the assistance of co-receptors such as endoglin and β-glycan (Goumans and Ten Dijke, 2018). Various proteins including noggin, chordin, follistatin, gremlin, coco, and cerberus act as ligand-traps to prevent TGF-β ligands from binding to receptors (David and Massague, 2018). Regulatory molecules such as FKBP12 and BAMBI inhibit the signaling pathway by docking at the cytoplasmic domain of TGF-β type I receptors (Wang et al., 1996; Onichtchouk et al., 1999).

The type II receptors phosphorylate the type I receptors to form a receptor complex, which then phosphorylates the receptor regulated SMADs (R-SMADs) intracellularly. Type I receptors for the TGFβ subfamily (ALK4, ALK5, and ALK7) mainly phosphorylate SMAD2 and SMAD3, whereas type I receptors for the BMP subfamily (ALK1, ALK2, ALK3, and ALK6) mainly phosphorylate SMAD1, SMAD5, and SMAD8. The activated R-SMADs form a complex with the central mediator SMAD4 and translocate into the nucleus, where it binds to specific gene loci under the guidance of signal-driven transcription factors (SDTFs) and lineage-determined transcription factors (LDTFs) as well as tripartite motif 33 (TRIM33) to regulate chromatin accessibility and gene transcription (Xi et al., 2011; David and Massague, 2018). A negative feedback loop of TGF-β signaling is mediated by the inhibitory SMADs: SMAD6 and SMAD7. SMAD7 can recruit E3 ubiquitin protein ligase SMURF2 to degrade the TGF-β receptor (Kavsak et al., 2000). SMAD6 not only interferes with the activation of SMAD2 phosphorylation by the receptor, but binds to R-SMAD and inhibits its binding to SMAD4 (Imamura et al., 1997; Hata et al., 1998). The TGF-β ligands can also signal through SMAD-independent pathways including the mitogen-activated protein kinase (MAPK) and phosphoinositide 3-kinase (PI3K) pathways (Derynck and Zhang, 2003; Figure 1).

It is intriguing that this seemingly simple “two-step” signal transduction of the TGF-β pathway has various and even opposite biological effects on a wide range of physiological processes, thereby reflecting the high spatiotemporal specificity of TGF-β signaling. The complexity of TGF-β signaling is manifested in the abundance and different combinations of its ligands, receptors and intracellular co-factors collaborating with SMADs. A single ligand can trigger multiple receptors [one of the 5 type II receptors (TGFBR2, BMPR2, ACVR2, ACVR2B, and AMHR2) in combination with one of the 7 type I receptors (ALK1-7)], and single receptor can interact with different ligands as well. The diversity of TGF-β ligand-receptor combinations leads to superimposed, synergistic or antagonistic effects on cells harboring different transcription factors and co-factors interacting with SMADs, resulting in complex biological effects (Massague, 2012).

### TGF-β Signaling in Cerebral Angiogenesis

The cerebrovascular network is developed via sprouting angiogenesis. The primary vessels of PNVP penetrate the CNS parenchyma and undergo remodeling to form a hierarchical vascular system composing of branched arteries and veins as well as capillaries, which is regulated by various signaling pathways including TGF-β signaling (Paredes et al., 2018).

#### Endothelial TGF-β-ALK5 Signaling in Sprouting Angiogenesis

Endothelial TGF-β signaling has been shown to be essential for cerebral angiogenesis, since Tgfbr2 or Alk5 gene knockout blood vessels fail to invade into the neuroepithelial layers and exhibit intracerebral hemorrhage (Nguyen et al., 2011). Genetic disruption of Smad4 in brain ECs leads to increased EC proliferation, impaired endothelial-pericyte interaction and intracerebral hemorrhage, providing a strong evidence that brain endothelial canonical TGF-β signaling plays essential roles in regulating brain angiogenesis and maintaining cerebrovascular integrity (Li et al., 2011).

A previous study has revealed the anti-angiogenic effect of TGF-β signaling in CNS vascular development (Arnold et al., 2014). Activated TGF-β signaling, by αVβ8 integrin, distributes as highest concentration in ventral brain regions and decreases in a gradient toward the dorsal brain regions, which is accompanied with stabilized vessels in ventral brain regions and greater vascular density, branch points and filopodia in dorsal brain region, suggesting that TGF-β signaling may play an anti-angiogenic role in cerebral angiogenesis. Consistently, loss of β8 integrin (Itgb8) or TGF-β1 or knockout of Alk5 or Tgfbr2 in ECs causes excessive vascular sprouting, branching and proliferation, which eventually leads to vascular dysplasia and cerebral hemorrhage (Arnold et al., 2014; Hirota et al., 2015). It has been further verified that neuroepithelial Itgb8 and endothelial neuropilin 1 (Nrp1) cooperatively promote cerebral angiogenesis by balancing TGF-β signaling. Endothelial Nrp1 inhibits β8 integrin activated TGF-β signaling to promote brain sprouting angiogenesis, and EC specific ablation of Nrp1 leads to increased levels of phosphorylated SMADs and embryonic lethality associated with defective sprouting angiogenesis and cerebral hemorrhage (Hirota et al., 2015).

Transforming growth factor-β signaling has also been shown to promote angiogenesis. TGF-β1 derived from radial glial cells promotes murine microcapillary brain EC migration and tube formation in vitro and stimulates cerebrovascular branching angiogenesis in the cerebral cortex, and this effect may be mediated by the balanced expression of pro-angiogenic gene GPR124 or anti-angiogenic gene, brain-specific angiogenesis inhibitor-1 (BAI-1) (Siqueira et al., 2018).

#### Endothelial BMP-ALK1 Signaling in the Stabilization of Brain Vessels

Bone morphogenetic protein-ALK1 signaling has been shown to limit EC number and maintain the quiescence of nascent vessels. BMP9 and BMP10 are physiological ligands of ALK1 during vascular development (Chen et al., 2013). In zebrafish, ALK1 functions in transducing hemodynamic forces into a biochemical signal which limits nascent vessel caliber (Corti et al., 2011). In mouse, ALK1 has been shown to mediate fluid shear stress by inducing BMP9 to inhibit endothelial proliferation and promote the recruitment of mural cells, thus maintaining vascular quiescence (Baeyens et al., 2016). Circulating BMP10 acts through endothelial ALK1 to activate pSMAD1/5/8 which decreases pro-angiogenic chemokine receptor cxcr4a expression and induces vasoconstrictive peptide endothelin 1 (Edn1), thereby limiting EC number and stabilizing nascent arterial caliber (Laux et al., 2013).

The mechanisms by which BMP-ALK1 regulates cerebrovascular development are quite limited; therefore, certain studies on the developmental mechanisms of mouse retinal vasculature can help us understand the related processes. The study using heterozygous Acvrl1+/- mice revealed that BMP9-ALK1 signaling inhibits EC proliferation and migration by activating PTEN to inhibit PI3K/Akt and MEK/ERK cascades, thereby maintaining retinal vascular quiescence (Alsina-Sanchis et al., 2018). Consistently, simultaneously silencing Bmp10 and Bmp9 in developing mice increases the retinal vascular density by promoting angiogenesis (Ricard et al., 2012).

#### TGF-β Signaling in the Formation and Maturation of BBB

Transforming growth factor-β signaling has been implicated in BBB formation and permeability by regulating tight and adherens junctions. The BBB is mainly composed of ECs which are characterized by the presence of tight and adherens junctions, and pericytes play an important role in the formation and maintenance of the BBB (Armulik et al., 2010; Bell et al., 2010; Daneman et al., 2010; Li et al., 2011). Endothelial TGF-β/SMAD4 signaling upregulates the adhesion molecule N-cadherin to facilitate the EC-pericyte interaction and BBB formation, in collaboration with Notch signal transduction (Li et al., 2011). Knockout of Smad4 in the brain ECs causes decreased expression of N-cadherin and pericyte detachment, leading to intraventricular hemorrhage and BBB breakdown during the perinatal period (Li et al., 2011). Besides, TGF-β1 derived from pericytes upregulates the expression of claudin-5 and promotes BBB maturation via decreasing endothelial CD146 expression (Chen et al., 2017).

Bone morphogenetic protein signaling has also been demonstrated to participate in the maintenance of BBB function. In zebrafish, BMP3 has been shown to regulate BBB integrity by promoting pericyte coverage (Lei et al., 2017). In rat cerebral vessel, BMP9/ALK1 signaling increases expression of endothelial transporters such as organic anion transporting polypeptide 1a4 at the BBB (Abdullahi et al., 2017). And BMP9/Alk1 is required for BBB stability, since ALK1 haploinsufficiency worsens the vascular leakage in diabetic mice. Mechanistically, ALK1 signaling inhibits VEGF-induced VE-cadherin phosphorylation and induces occludin expression, thereby enhancing the BBB function (Akla et al., 2018).

#### Non-endothelial TGF-β Signaling in Cerebral Angiogenesis

Cerebral angiogenesis is not only programmed in ECs, but also orchestrated by dynamic TGF-β signaling in other cell types within or outside the NVU, including pericytes, astrocytes, oligodendrocyte precursor cells, neural progenitors, preosteoblasts and periosteal dura cells.

Brain pericytes have been shown to induce and upregulate the functions of BBB through continuous TGF-β production (Dohgu et al., 2005). Pericyte ALK5 upregulates tissue inhibitor of matrix metalloproteinase 3 (TIMP3) to control endothelial morphogenesis in the germinal matrix. Specific knockout of Alk5 in embryonic mouse pericytes causes degradation of the basement membrane by upregulated matrix metalloproteinases (MMPs), resulting in severe germinal matrix hemorrhage-intraventricular hemorrhage (GMH-IVH) (Dave et al., 2018).

Astrocytes, whose endfeet interact with ECs of the neural capillaries, play a critical role in cerebral angiogenesis and BBB formation though BMP signaling. Targeted disruption of BMP type IA receptor (BMPR1A) in telencephalic neural stem cells leads to upregulated expression of VEGF in mutant astrocytes, impaired EC-astrocyte interaction, and cerebrovascular malformation, demonstrating that BMP signaling in astrocytes is essential for a functional BBB (Araya et al., 2008). A very recent study showed that BBB breakdown in aging humans and rodents is associated with hyperactivation of TGF-β signaling in astrocytes. Conditional genetic knockdown of astrocytic TGF-β receptor-coding genes or pharmacological inhibition of TGF-β signaling rescues the phenotypes in aged mice (Senatorov et al., 2019).

Oligodendrocyte precursor cells have also been shown to maintain BBB integrity through TGF-β signaling. TGF-β1 derived from oligodendrocyte progenitor cells can activate the MEK/ERK signaling pathway in ECs to promote tight junction protein expression and improve BBB integrity, and knockout of Tgfbr1 in oligodendrocyte progenitor cells leads to cerebral hemorrhage and disruptive BBB in mice (Seo et al., 2014).

Several neural progenitors have also been shown to play important roles in brain region-specific angiogenesis via TGF-β signaling. Tgfbr2 silencing in forebrain-derived neural progenitors and neural cells impedes EC migration and sprouting, decreases vessel density and branching via altered secretion of pro- and anti-angiogenic factors, thereby leading to intracerebral hemorrhage in the telencephalon (Hellbach et al., 2014). Neural progenitor S1P signaling regulates integrin β8 gene expression, thereby activating local TGF-β signaling that promotes germinal matrix vasculature development. Disruption of S1P signaling in neural progenitors results in defective angiogenesis and hemorrhage, as well as phenotypes mimicking the germinal matrix hemorrhage in humans (Ma et al., 2017).

In addition, BMP2 and BMP4 derived from preosteoblasts and periosteal dura are essential for dural cerebral vein formation. Loss of Twist1 or BMP2/4 signaling in skull progenitor cells and dura leads to cerebral vein malformations, similar to that in humans with craniosynostosis (Tischfield et al., 2017).

### TGF-β Signaling in Endothelial-to-Mesenchymal Transition (EndMT)

Endothelial-to-Mesenchymal Transition is a complex biological process and mainly refers to the trans-differentiation of ECs into mesenchymal stem cells, fibroblasts, SMCs or pericytes (Dejana and Lampugnani, 2018). During the process of EndMT, ECs lose the expression of endothelial markers (such as CD31 and VE-cadherin), and exhibit increased expression of mesenchymal transcription factors and molecular markers [such as Snail1, Slug (Snail2), Twist, ZEBs, vimentin, α-SMA, fibroblast-specific protein-1 (FSP-1; also known as S100A4 protein), fibroblast activating protein (FAP), and fibrillary collagens type I and type III] to obtain a mesenchymal morphology. Mesenchymal cells derived from EndMT gain enhanced ability of cell migration and invasion via disturbing the paracellular connection and polarity of ECs.

Activation of the TGF-β signaling pathway is the most important onset of EndMT (Ma et al., 2020). All three TGF-βs (TGF-β1, TGF-β2, and TGF-β3) have been shown to induce EndMT, while TGF-β2 seems to be more effective than TGF-β1 or TGF-β3 (Sabbineni et al., 2018). TGF-β ligands activate the TGFBR2 and ALK2 or ALK5 in ECs, and induce the pSMAD2/3/4 complex to translocate into the nucleus, where they interact with other transcription factors required for EndMT including Snail1, Snail2, Zeb1, Zeb2, KLF4, TCF3, and Twist and subsequently trigger the expression of mesenchymal transcription factors and molecular markers. TGF-β ligands also trigger EndMT through the non-canonical TGF-β pathways including MAPK, PI3K, and RhoA pathways (Piera-Velazquez and Jimenez, 2019).

Emerging studies have revealed that BMP signaling serves as a gatekeeper by antagonizing TGF-β-induced EndMT in ECs. BMP7 has been shown to inhibit hypoxia-induced EndMT and gremlin-1-mediated EndMT (Zhang et al., 2018, 2020). Loss of Bmpr2 in ECs leads to EndMT characterized by conversion of VE-cadherin to junctional N-cadherin, Slug and Twist upregulation, as well as increased expression of extracellular matrix (ECM) proteins (Hiepen et al., 2019). BMPR2-JNK signaling axis has also been shown to antagonize inflammation-induced EndMT (Sanchez-Duffhues et al., 2019).

Physiologically, EndMT plays essential roles during cardiovascular development, such as angiogenic sprouting and cardiac valve formation (Kruithof et al., 2012; Welch-Reardon et al., 2014). Dysregulation of EndMT has been associated with pathological situations, such as malignant diseases, fibrotic disorders and vascular diseases (Piera-Velazquez and Jimenez, 2019).

Emerging evidence indicates that dysregulated EndMT contributes to certain cerebrovascular diseases (Piera-Velazquez and Jimenez, 2019). The first evidence that EndMT is involved in the pathological process of cerebrovascular diseases was from the study of cerebral cavernous malformation (CCM). TGF-β signaling mediated EndMT is a direct cellular mechanism leading to CCMs in either mouse models or human patients (Maddaluno et al., 2013; Cuttano et al., 2016). Shortly after, another study reported that a meningeal pathogen Group B Streptococcus infection induces Snail1 expression and endothelial dedifferentiation, leading to BBB disruption, suggesting that EndMT might also contribute to BBB deficiency (Kim et al., 2015). Very recently, several studies have revealed that EndMT occurs in multiple sclerosis (MS), ischemic stroke, as well as brain arteriovenous malformations (AVMs) in humans (Derada Troletti et al., 2019; Chen et al., 2020; Shoemaker et al., 2020). All these results indicate that dysregulated EndMT might be an important pathological process involved in a variety of cerebrovascular disorders. However, the causal link between EndMT and various cerebrovascular diseases needs to be further established.

## Dysregulation of TGF-β Signaling in Cerebrovascular Diseases

Recent studies have shown that defects in TGF-β signaling are associated with human cerebrovascular diseases. Pathogenic mutations in TGF-β signaling, such as ENG, ALK1 gene mutations, are associated with type 1 and type 2 hereditary hemorrhagic telangiectasia (HHT), as well as Loeys-dietz syndrome with cerebrovascular events (McAllister et al., 1994; Cunha et al., 2017; Laterza et al., 2019). Some genome-wide association studies (GWAS) or whole exome trio sequencing have uncovered various pathogenic gene variants in the TGF-β pathway, which are associated with small vessel ischemic strokes, intracerebral hemorrhages and sporadic brain AVMs (Weinsheimer et al., 2016; Yilmaz et al., 2017; Wang et al., 2018; Chung et al., 2019). Increased expression of TGF-β1 has been found in the brain tissue after ischemic stroke, as well as in hereditary cerebral hemorrhage with amyloidosis-Dutch type (Krupinski et al., 1996; Grand Moursel et al., 2018), while a recent transcriptome-wide RNA sequencing study revealed that TGF-β signaling was downregulated in patients with brain AVMs (Hauer et al., 2020). All these evidences suggest that dysregulation of TGF-β signaling may contribute to the onset and progression of cerebrovascular diseases. While there are not many studies on the mechanisms of cerebrovascular diseases related to TGF-β dysfunction, we discuss the three most studied cerebrovascular diseases caused by dysregulation of TGF-β signaling.

### Cerebral Cavernous Malformation (CCM)

Cerebral cavernous malformation is a cerebrovascular disease causing recurrent cerebral hemorrhage, headaches, seizures and stroke, which is histologically characterized by clusters of dilated vascular sacs with ECs lacking tight junctions and mural cell coverage (Goldstein and Solomon, 2017; Stapleton and Barker, 2018). Genetically, CCMs can be categorized into familial and sporadic types. Approximately, 20% of all CCMs are Familial CCMs which present autosomal dominant inheritance with loss-of-function germline mutations in any one of the following three genes: *CCM1/KRIT1*, *CCM2/malcavernin*, or *CCM3/PDCD10* (Zafar et al., 2019). The sporadic CCMs are non-hereditary and are probably caused due to somatic mutations of CCM genes (McDonald et al., 2014).

In both familial and sporadic CCM patients, TGF-β signaling is activated during pathological progression, as indicated by nuclear accumulation of endothelial pSMAD3 accompanied by expression of EndMT markers in lesions of familial and sporadic cavernomas (Maddaluno et al., 2013; Bravi et al., 2016). Besides, Kruppel-like factor 2 (KLF2) and KLF4, the activators of the BMP signaling, are significantly upregulated in ECs of familial and sporadic CCM lesions (Cuttano et al., 2016; Zhou et al., 2016). Activated TGF-β/BMP signaling has also been observed in cultured cells wherein all three Ccm genes were knocked down in ECs (Maddaluno et al., 2013; Cuttano et al., 2016), especially under low fluid shear stress conditions (Li et al., 2019).

**FIGURE 2:**
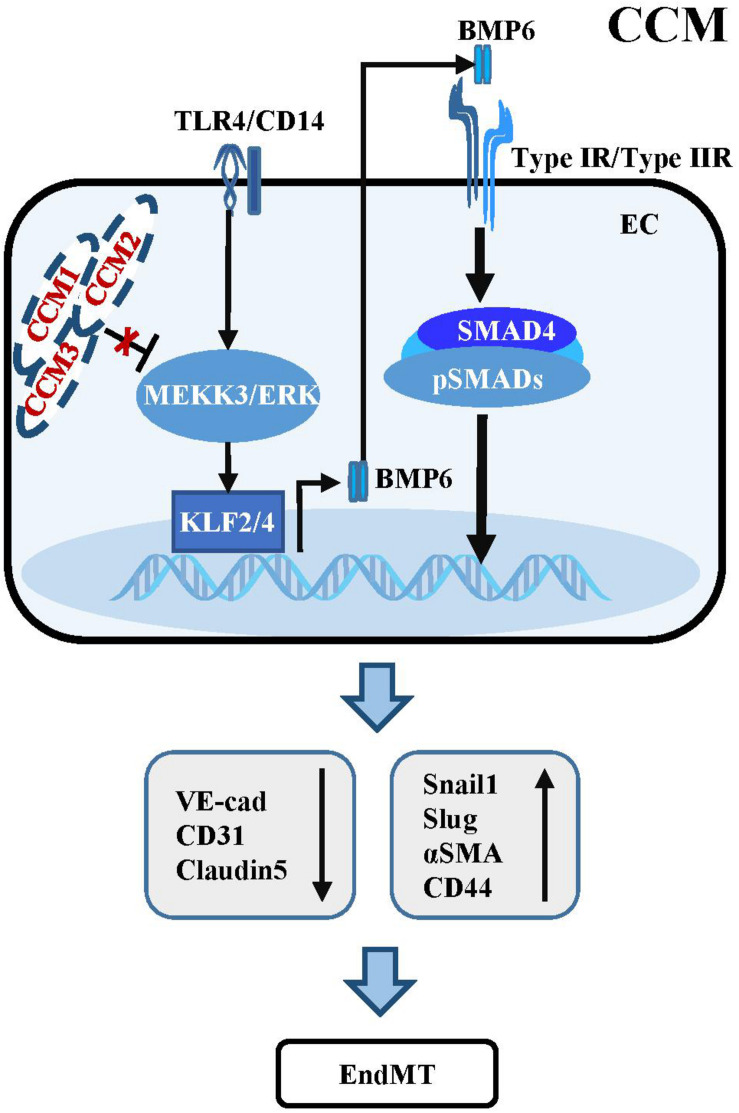
Dysregulation of TGF-β/BMP signaling in CCM. Mutant CCM release the inhibition of MEEK3/ERK pathway, which trigger the expression of KLF2/4. KLF2/4 subsequently suppress the expression of endothelial markers and induce the expression of EndMT-related molecules. KLF4 could also transcriptionally upregulate BMP6 to active TGF-β/BMP cascades in ECs. Besides, activation of TLR4 associated with CD14 by Gram-negative bacteria and lipopolysaccharide injection could increase the expression of Klf2/4 and promote CCM formation.

The activation of TGF-β/BMP signaling has been confirmed in endothelial specific Ccm1 and Ccm3 knockout mice. Ablation of Ccm1 in ECs activates the expression of endogenous Bmp6 which induces the upregulation of pSMAD1 and pSMAD3 and triggers EndMT resulting in cerebral vascular malformations (Maddaluno et al., 2013). The upregulation of Bmp6 caused by mutant Ccm1 could be mediated by KLF4 which directly binds to the promoters of Bmp6 and some EndMT markers to induce their expression (Cuttano et al., 2016; Figure 2). Moreover, increased levels of pSMAD1 and pSMAD3 were observed in ECs of endothelial Ccm3 knockout mice (Bravi et al., 2015). Small-molecule inhibitors of TGFBR, pSMAD or BMP signaling could prevent EndMT and reduce the size and number of cerebral malformations, demonstrating that dysregulation of TGF-β/BMP signaling directly contributes to the onset and pathological process of CCMs (Maddaluno et al., 2013).

Some studies have uncovered the causal function of mitogen-activated protein kinase kinase Kinase 3 (MEKK3) and KLF2/4 in CCM pathogenesis, which is independent of TGF-β/SMAD signaling (Zhou et al., 2016). Endothelial-specific disruption of Mekk3, Klf2 or Klf4 significantly suppresses CCM and rescues the lethal phenotype in Ccm2 mutant mice. Consistently, the levels of KLF2 and KLF4 are increased in ECs of lesions in familial and sporadic CCM patients (Cuttano et al., 2016; Zhou et al., 2016). Supportively, ponatinib, a small-molecule compound inhibits MEKK3 activity to increase expression of the downstream Klf gene, suppresses CCM in neonatal Ccm1 deficient mouse models (Choi et al., 2018). In addition, activation of TLR4 by Gram-negative bacteria and lipopolysaccharide injection could increase the expression of Klf2/4 and promote CCM formation in Ccm1 and Ccm2 knockout mice (Tang et al., 2017; Figure 2). These inconsistencies with respect to the role of TGF-β signaling in the development and progression of CCM might be largely due to the different genetic backgrounds of the mouse models used, and the different stages of CCM pathogenesis analyzed in different experiments. Additional genetic rescue experiments might be helpful to further demonstrate the causal link between dysregulation of TGF-β signaling and the development and progression of CCM.

### Hereditary Hemorrhagic Telangiectasia (HHT)

**FIGURE 3:**
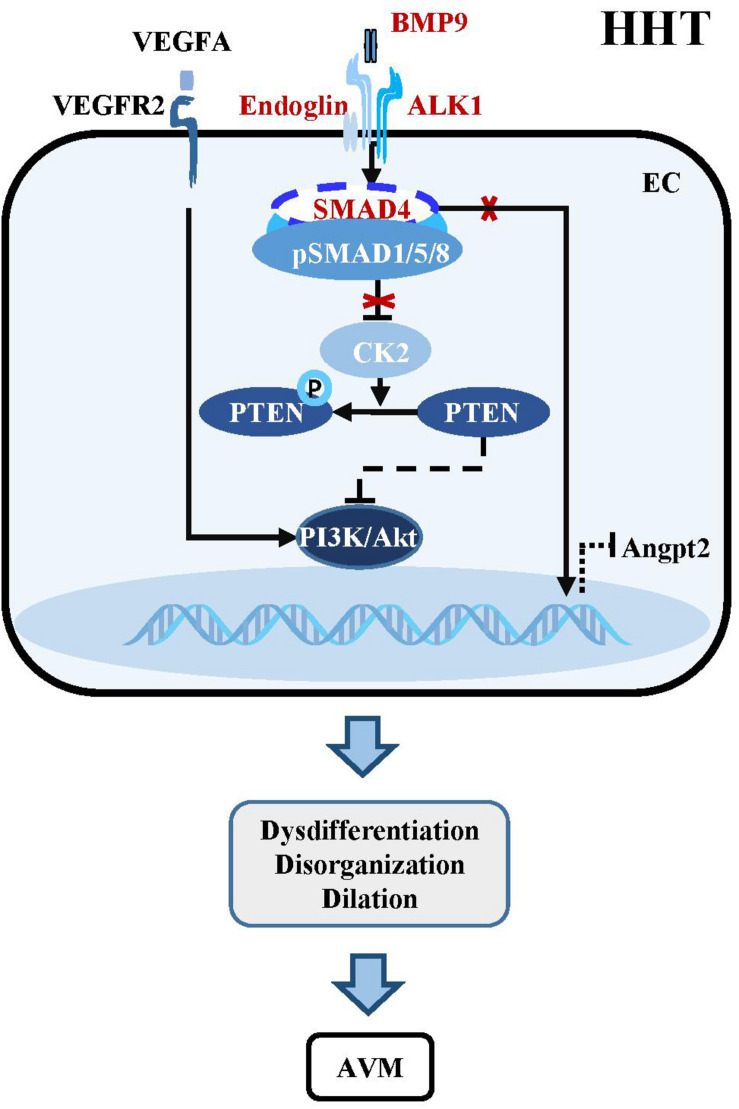
Dysregulation of BMP-ALK1-Smad4 signaling in HHTs. The mutant components within the BMP-ALK1 pathway including BMP9, ALK1, Endoglin, and SMAD4 are unable to suppress the transcription of CK2. Therefore, PTEN is phosphorylated by CK2 and loss the capacity to inhibit the PI3K/Akt pathway, which result in dysdifferentiation, disorganization, dilation and subsequent AVMs in cerebrovasculatures. Besides, AVMs-caused hypoxia increases the level of VEGFA, which subsequently binds VEGFR2 to active downstream PI3K/Akt signaling to aggravate the pathological process of AVMs. In addition, mutant SMAD4 increases the expression of angiopoietin-2 (Angpt2), leading to AVMs.

Hereditary hemorrhagic telangiectasia, also known as Osler-Weber-Rendu syndrome, is an autosomal dominant genetic disorder characterized as telangiectasia and AVMs affecting vessels in multiple organs and tissues including the brain (Brinjikji et al., 2015; Kritharis et al., 2018). Five types of HHT have been described, and HHT1 and HHT2 contribute to the disease in more than 80% of patients with definite HHT (Brinjikji et al., 2015). Some HHT patients display brain AVMs, often accompanied by cerebral hemorrhage, seizure, headache, or focal neurologic symptoms (Brinjikji et al., 2017a, b). Genetic screening of HHT patients has identified four mutated genetic loci, all of which are involved in the TGF-β signaling pathway, including BMP9 ligand encoding gene *GDF2* (HHT5 or HHT like), type I receptor ALK1 encoding gene *ACVRL1* (HHT2), co-receptor endoglin encoding gene *ENG* (HHT1) and intracellular mediator SMAD4 encoding gene *MADH4* (JP-HHT) (McAllister et al., 1994; Johnson et al., 1996; Gallione et al., 2004; Wooderchak-Donahue et al., 2013; Figure 3).

BMP9/10-ALK1 signaling suppresses HHTs through SMAD-dependent or SMAD-independent pathways. Endothelial-specific knockout of Alk1 triggers cerebral AVMs mimicking the pathologic characteristics of HHT (Park et al., 2009). In adult mouse, combined with VEGF stimulation, knockout of Alk1 could alter cerebral arteriovenous molecule specificity and induce AVMs (Walker et al., 2011). Zebrafish harboring mutations in Bmp9 and duplicate Bmp10 paralogs, Bmp10 and Bmp10-like exhibit cranial AVMs mimicking Acvrl1 mutants (Capasso et al., 2020). In postnatal retina, BMP9/10 ligand blockade and endothelial-specific homozygous ALK1 inactivation induces excessive angiogenesis via activating VEGF and PI3K/Akt signaling (Ola et al., 2016; Ruiz et al., 2016; Alsina-Sanchis et al., 2018). Pharmacological or genetic inhibition of PI3K rather than VEGFR could abolish ALK1-induced vascular hyperplasia in vivo, confirming that PI3K/Akt is the core mechanism downstream of BMP9/10-ALK1 signaling in maintaining vascular quiescence (Alsina-Sanchis et al., 2018; Ola et al., 2018; Iriarte et al., 2019; Figure 3).

Mice with homozygous or heterozygous deletion of Eng with VEGF treatment exhibit brain AVMs (Choi et al., 2012, 2014), and endothelial-specific Eng knockout mice spontaneously develop AVMs in the retina or brain (Mahmoud et al., 2010; Choi et al., 2014). In cerebral and retinal vessels, the Eng-null ECs cannot migrate against blood flow toward the arteries, leading to the accumulation and proliferation of ECs thereby triggering AVMs. Increased VEGFA expression which activates PI3K/Akt signaling through VEGFR2 may be responsible for stimulating sprouting angiogenesis and promoting venous differentiation in Eng mutant mice (Jin et al., 2017). Consistently, a recent study showed that ECs lacking Eng exhibit increased VEGF sensitivity and abnormal proliferation resulting in the formation of peripheral AVM (Tual-Chalot et al., 2020).

The essential role of endothelial SMAD4 in the maintenance of cerebrovascular integrity has been demonstrated by the study using a brain endothelial specific Smad4 knockout mouse, which develops phenotypes partially simulating HHT patients, such as dilated vessels, increased EC proliferation, intracranial hemorrhage and BBB breakdown (Li et al., 2011). Postnatally inducible endothelial Smad4 knockout results in AVM in neonatal and adult mice, which is comparable with the phenotypes observed in inducible endothelial Alk1 and Eng knockout mice (Crist et al., 2018; Kim et al., 2018; Ola et al., 2018). Mechanistically, SMAD4-mediated BMP9/10-ALK1 signaling inhibits the transcription of casein kinase 2 (CK2) which limits PTEN phosphorylation and PI3K/Akt activation, thereby preventing AVMs in the brain, retina, and gastrointestinal tract (Ola et al., 2018). In addition, Smad4 knockout leads to increased angiopoietin-2 (Angpt2) expression in ECs, which might cause AVM by changing the size and shape of ECs in the retina of Smad4 mutant mice (Lan et al., 2007; Crist et al., 2019; Figure 3).

These studies based on mouse models that mimic human HHT patients have provided the causal link between dysregulated TGF-β signaling and the pathogenesis of HHT. Blood flow stimulates BMP9-ALK1-ENG-SMAD4 signaling to maintain EC quiescence by suppressing EC proliferation and inducing pericyte recruitment (Baeyens et al., 2016), which involves PI3K/Akt signaling, Angpt2 signaling and possibly other factors (Alsina-Sanchis et al., 2018; Ola et al., 2018; Crist et al., 2019). It is worth noting that AVMs develop due to a combination of gene mutations in TGF-β signaling with angiogenic induction (via VEGF stimulation or wounding) (Park et al., 2009; Garrido-Martin et al., 2014), supporting the “Two hit mechanism” in HHT. Consistently, the tissues that are most vulnerable to AVMs or telangiectasia are those-susceptible to repeated damage and repair, such as the face, lips, and fingers in HHT patients (Brinjikji et al., 2015). Further investigation is required to elucidate whether dysregulation of other signaling pathways which cross talk with the TGF-β signaling pathway could serve as the second hits in the pathogenesis of HHT.

### Cerebral Autosomal Recessive Arteriopathy With Subcortical Infarcts and Leukoencephalopathy (CARASIL)

Cerebral Autosomal Recessive Arteriopathy with Subcortical Infarcts and Leukoencephalopathy is a rare autosomal recessive cerebrovascular disease that mainly occurs in cerebral white matter and basal ganglia, causing early adult-onset dementia, gait disturbance, alopecia, and low back pain (Nozaki et al., 2014; Tikka et al., 2014). Histologically, CARASIL displays cerebral arteriopathy showing fibrous proliferation of intima, loss of vascular SMCs and thickening of meningeal and parenchymal arteries. Fibrous hyperplasia in arteries results in the impaired contraction, leading to subcortical lacunar infarcts and subsequent vascular dementia (Oide et al., 2008; Ito et al., 2016). Genetically, mutations in high-temperature requirement serine peptidase A1 (*HTRA1*) gene have been identified to be associated with CARASIL (Hara et al., 2009). In patients, TGF-β1-pSMAD2 activation was observed in cerebral small arteries (Hara et al., 2009; Shiga et al., 2011).

The mechanistic role of TGF-β signaling in the pathogenesis of CARASIL, is a debatable topic. HRTA1 is a serine protease which is strongly expressed in ECs, vessel SMCs and pericytes (De Luca et al., 2003). HtrA1 knockout mice display a significantly decreased retinal vascular density which coincides with patients presenting reduced cerebral small vessels (Zhang et al., 2012). Mechanistically, HTRA1 cleaves the pro-domain of proTGF-β1 and TGF-β receptors to antagonize TGF-β signaling (Oka et al., 2004; Shiga et al., 2011; Graham et al., 2013). Consistently, HtrA1 knockout either in vivo or in cultured cells induces the expression of TGF-β ligands and activates pSMAD2/3 signaling (Zhang et al., 2012; Klose et al., 2019). All these results indicate that abnormal activation of TGF-β signaling contributes to the pathogenesis of CARASIL. However, there are studies showing that impaired TGF-β signaling is involved in CARASIL pathogenesis (Beaufort et al., 2014; Fasano et al., 2020). Loss of HtrA1 leads to defective HTRA1-mediated LTBP-1 processing and reduced TGF-β signaling (Beaufort et al., 2014), and fibroblasts derived from *HTRA1* mutation carriers exhibit no significant change in pSMAD2/3 expression (Fasano et al., 2020). The possible reasons for the discrepancy might partially be due to different *HTRA1* mutations leading to different outcomes (Verdura et al., 2015; Lee et al., 2018). Future studies should identify the natural characteristics of CARASIL associated mutations and develop animal models that accurately mimic all pathological and molecular aspects of human CARASIL patients, which will help uncover the mechanisms of CARASIL and discover new therapeutic targets.

## Potential Therapies Targeting TGF-β Signaling

Current therapies for CCM, HHT, and CARASIL patients mainly rely on surgery or relieving complications (Tikka et al., 2014; Kritharis et al., 2018; Stapleton and Barker, 2018). Recent advances in understanding the mechanisms of dysfunctional TGF-β signaling which results in cerebrovascular diseases has provided hope to develop pharmacological and genetic therapies for these diseases.

Activated TGF-β/BMP signaling has been demonstrated to contribute to the onset and progression of CCMs in patients and mouse models (Maddaluno et al., 2013; Bravi et al., 2016; Cuttano et al., 2016). Therefore, it is expected that therapeutics targeting TGF-β/BMP signaling would be beneficial for CCMs. Indeed, TGFBR1/pSMAD inhibitors LY364947 and SB431542 as well as BMPR1 inhibitor dorsomorphin (DMH1) strikingly reduce the level of pSMAD1 and pSMAD3, prevent the expression of EndMT markers, and decrease the number and size of vascular malformation lesions in CCM1 mutant mice (Maddaluno et al., 2013). KLF4 has been shown to be a good therapeutic target for CCM. Ccm1 knockout results in MEKK3-MEK5-ERK5-MEF2 signaling dependent activation of KLF4 which promotes the expression of Bmp6. A specific MEK5 inhibitor BIX-02189 (Tatake et al., 2008) significantly decreases pERK5 and KLF4 expression, inhibits Bmp6 upregulation and EndMT in CCM1 deficient ECs (Cuttano et al., 2016), indicating that inhibitors of the MEKK3-MEK5-ERK5-MEF2 axis might be useful for suppressing BMP signaling and EndMT in the pathogenesis of CCM. There are several novel drugs targeting TGF-β signaling, developed through preclinical trials and further tested in clinical trials, including anti-ligand antisense oligonucleotides (ASOs), ligand-competitive peptides, antibodies targeting ligands, receptors or associated proteins, and inhibitors against TGF-β receptor kinases for various diseases (Akhurst and Hata, 2012; Graham et al., 2013; Kemaladewi et al., 2014; Aykul and Martinez-Hackert, 2016; Wu et al., 2017; Holmgaard et al., 2018). It is worth examining whether these candidate drugs that target TGF-β signaling could inhibit the progress of CCMs.

The majority of HHT patients have pathogenic loss of function mutations in TGF-β signaling. Although many studies have uncovered the molecular mechanisms underlying HHTs caused by dysfunctional TGF-β signaling, there is currently no efficient drug for HHT treatment. Current drug therapy regimens mainly focus on interfering with the downstream core signaling pathway such as activated VEGF and PI3K/Akt signaling (Alsina-Sanchis et al., 2018; Ola et al., 2018). Since haploinsufficiency of endoglin and ALK1 have been identified as the causes of HHT1 and HHT2, a better understanding of the regulation of their expression levels at the transcriptional level or post-transcriptional level will help developing therapeutic strategies targeting endoglin and ALK1 expression or function. Indeed, a very recent study shows that ALK1-overexpression could normalize SMAD and NOTCH target gene expression, restore the effect of BMP9 on suppression of p-Akt, and inhibit the development of AVMs in Alk1- and Eng-inducible knockout mice, suggesting that ALK1 overexpression or activation might be a potential therapeutic strategy for HHT patients (Kim et al., 2020).

Genome editing may serve as the final solution. CRISPR-based genome editing has been demonstrated as a powerful tool for treating genetic diseases (Pickar-Oliver and Gersbach, 2019). The CRISPR-Cas9 system has been demonstrated to efficiently correct gene mutations in various mouse models of human diseases, including cataracts, muscular dystrophy and many others (Wu et al., 2013; Long et al., 2014). Recent studies show that base editing can correct mutations in human cells and in a mouse model of genetic deafness, and a newly developed template-free Cas9 editing is able to precisely correct the pathogenic mutations in human cells (Gaudelli et al., 2017; Gao et al., 2018; Shen et al., 2018). A newly developed CRISPR-CasΦ system, with a molecular weight which is only half of Cas9 or Cas12a displays expanded target recognition capabilities and is functional in human cells as well (Pausch et al., 2020), providing a new genome editing tool for treating cerebrovascular diseases. Once the causal link between mutations in TGF-β signaling and cerebrovascular diseases has been established, genome editing will likely correct these mutant genes to heal the related cerebrovascular diseases.

## Conclusion and Perspectives

Previous studies have demonstrated the crucial function of TGF-β signaling in cerebral vasculature development and integrity, and uncovered the causal link between the dysfunctional TGF-β signaling and the onset or progression of several cerebrovascular diseases such as CCM, HHT and CARASIL. However, the related mechanisms underlying the dysregulation of TGF-β signaling resulting in cerebrovascular diseases remains to be further elucidated. In recent years, using the rapidly developed single cell sequencing technology and advanced graphics algorithm, researchers have revealed the unappreciated heterogeneity and plasticity of human and mouse cerebral blood vessels, discovering not only new markers for different subtypes of ECs but also a new cell type adjacent to the blood vessel (Schaum et al., 2018; Vanlandewijck et al., 2018; Kalucka et al., 2020). Further investigation of the role and mechanism of TGF-β signaling in the regulation of cerebrovascular heterogeneity and plasticity will help to understand the function of TGF-β signaling in the occurrence and development of cerebrovascular diseases.

There are not many animal models that can accurately mimic the genetic and pathological characteristics of human cerebrovascular diseases. Rapid advances in genome editing technologies based on CRISPR-Cas systems provide powerful tools for generating animal models carrying genomic mutations precisely mimicking the ones in human patients (Pickar-Oliver and Gersbach, 2019). Studies using cell lineage tracing technology combined with single cell sequencing in animal models of human cerebrovascular diseases will help reveal the cellular and molecular mechanisms of cerebrovascular diseases and discover new therapeutic targets. In addition, human cortical organoids with functional cerebral vessels will provide valuable models for dissecting the roles of TGF-β signaling in the development and progression of human cerebrovascular diseases (Cakir et al., 2019).

Although recent advances have indicated that targeting TGF-β signaling will be a potential strategy for the treatment of cerebrovascular diseases, clinical transformation is still challenging. Considering the cell context-dependent pleiotropic roles of the TGF-β signaling pathway, the selectivity and dosage of targeted drugs may be crucial for the desired therapeutic effects. Previous research has identified various TGF-β inhibitory drugs involving almost every level in the TGF-β signaling cascade, some of which have been proved safe and effective for treating systemic sclerosis, cancers or idiopathic pulmonary fibrosis in clinical trials (Rice et al., 2015; Yingling et al., 2018; Joyce et al., 2019; Kelley et al., 2019; Papachristodoulou et al., 2019; Santini et al., 2019). These existing TGF-β inhibitory drugs provide potential therapeutic opportunities for treating cerebrovascular diseases with activated TGF-β signaling. For cerebrovascular diseases with loss-of-function mutations in TGF-β signaling, somatic genome editing may provide tools to correct the mutations or enhance TGF-β signaling.

Increased clinical research data shows that there is a close correlation between cerebrovascular and central nervous system diseases. Abnormal cerebrovascular structure and function are closely related to brain atrophy, dementia and various neurodegenerative disorders and cognitive impairment (Turner et al., 2016; Yang et al., 2017; Iadecola and Gottesman, 2018; Kummer et al., 2019). Dysregulated TGF-β signaling has been observed in neurodegenerative diseases accompanied by cerebrovascular abnormalities (von Bernhardi et al., 2015). Further studying the synergistic mechanisms by which TGF-β signaling maintains the homeostasis of the cerebrovascular and central nervous system might be very helpful in uncovering the direct causal link between cerebrovascular and central nervous system diseases, providing new theoretical basis and treatment strategies for joint preventing and treating cerebrovascular and central nervous system diseases.

## Author Contributions

YZ and XY wrote the review. All authors listed have made a substantial, direct and intellectual contribution to the work, and approved it for publication.

## Conflict of Interest

The authors declare that the research was conducted in the absence of any commercial or financial relationships that could be construed as a potential conflict of interest.
